# Role of lactoferrin in osteopenia and osteoporosis

**DOI:** 10.3389/fnut.2025.1648510

**Published:** 2025-09-03

**Authors:** Deming Li, Maofeng Gao, Meng Li, Huan Zhao, Xichao Zhou, Qiaoli Gu, Huilin Yang, Qin Shi

**Affiliations:** Department of Orthopedics, The First Affiliated Hospital of Soochow University, Orthopedic Institute of Soochow University, Suzhou Medical College of Soochow University, Suzhou, Jiangsu, China

**Keywords:** lactoferrin, osteopenia, osteoporosis, bone metabolism, mechanisms

## Abstract

Osteopenia and osteoporosis (OP) are serious public health concerns that impose substantial health and economic burdens on the global population. Lactoferrin (Lf) is a natural iron-binding glycoprotein that exhibits numerous biological functions. This review summarized the role of circulating Lf and related biomarkers in maintaining bone health. Lf may protect against OP through various mechanisms, including the osteoprotegerin/receptor activator of nuclear factor κB ligand/receptor activator of nuclear factor κB, bone morphogenetic protein signaling pathway, liver–bone axis, insulin-like growth factor 1 signaling pathway, autophagy, and gut microbiota. Moreover, the peptides derived from Lf and Lf-based nanoformulations or biomaterials show potential in preventing OP. Overall, this review supports the potential application of Lf for OP.

## Introduction

Osteopenia and osteoporosis (OP) refer to conditions characterized by decreased bone mineral density (BMD); however, they differ in the severity of bone loss. Osteopenia represents a milder form of bone loss than OP. The global prevalence of osteopenia and OP is estimated to reach 40.4% and 19.7%, respectively (1), resulting in a substantial health and financial burden. OP can lead to fragile fractures, which can lead to disability and even death in older adults (2, 3).

Lactoferrin (Lf) is primarily found in milk. It is a natural iron-binding glycoprotein with a molecular weight of approximately 78 kDa and consists of >690 amino acids. Lf supplementation has been reported to be beneficial against various diseases, including obesity, type 2 diabetes, atherosclerosis, non-alcoholic liver disease, alcoholic liver disease, and some cancers (4, 5). Studies have reported the positive effect of Lf on OP. To obtain a comprehensive understanding of the association between Lf and OP, this review summarizes the protective effects and underlying mechanisms of Lf treatment in OP.

## Association between Lf and bone health

Endogenous Lf is present in serum, neutrophils, and saliva. Although reference values for serum Lf levels have not been established in the general population, most studies have indicated circulating Lf concentrations of ~500 ng/ml (6–10); however, some studies have reported that Lf circulates at concentrations as low as 500 pg/ml or as high as 3,500 ng/ml (11, 12). Lf levels in biological samples are associated with several diseases, including inflammatory bowel disease (13), Alzheimer's disease, allergic rhinitis (9), and rheumatoid arthritis (11). Lf is a potential biomarker for these diseases; however, studies on the association between serum Lf and bone health are limited, and their interactions remain unclear. In a cross-sectional study, although there was no association between serum Lf concentration and BMD or N-terminal propeptide of type 1 precollagen (P1NP), a positive correlation of Lf with parathyroid hormone and β-crosslaps (β-CTx) was observed in older women (14). Specifically, circulating Lf was associated with bone resorption markers (14). Hanna et al. evaluated the saliva Lf levels in patients with OP and in healthy controls without OP (15). The results indicated that, although not statistically significant, Lf levels decreased in both unstimulated and stimulated saliva from OP patients compared with the control group (15); however, this was a preliminary analysis with no adjustments performed. The predictive value of Lf in OP still requires investigation through large-scale studies.

Studies on the effects of exogenous Lf supplementation for OP have been conducted primarily in cells or animals. Although Bharadwaj et al. found that a milk ribonuclease-enriched Lf supplement could restore the balance of bone turnover within a short period in postmenopausal women (16), the study failed to isolate Lf and report BMD. Therefore, direct evidence from clinical trials remains lacking. Low research priority and limited market attention might be two important reasons. On the one hand, more studies on OP mostly focused on bisphosphonates, denosumab, and hormonal therapy (17), and Lf was regarded as a relatively low priority in scientific resource allocation. On the other hand, Lf had a smaller market scale than medicines or other classical nutrients (*e.g.*, 247 million for Lf in 2025 *vs*. 1.3 billion for vitamin D in 2022), resulting in insufficient support for clinical trials (18, 19). Moreover, cohort studies on the associations between Lf intake and BMD were also hard to conduct. Since almost all Lf intake was from milk, Lf intake inevitably coincided with increased calcium intake. Consequently, even though calcium intake could be adjusted to some extent by statistical methods, the confounding factor cannot be entirely eliminated.

Endogenous Lf is also present in breast milk, saliva, and neutrophils (4, 20). Immunohistochemical analyses have indicated that fetal osteoblasts (OBs) exhibit Lf immunoreactivity, whereas adult OBs do not (21, 22). In the fetus, Lf was detected up to the 18th week of gestation and disappeared after the 30th week (22, 23). Thus, Lf may be involved in bone growth regulation during the early phases (23) but not as an optional biomarker for OP in adults. In addition, Lf may be expressed in osteocartilagineous tumors, chondroblastomas, chondromyxoid fibromas, and osteoid osteomas but not in osteosarcomas, chondrosarcomas, ossifying fibromas, osteochondroma, and enchondromas, which may reflect a mature phenotype of these tumors (21, 23, 24).

## Effects of Lf on osteoblasts and osteoclasts

Bone remodeling is tightly regulated through crosstalk between bone-forming OBs and bone-resorbing osteoclasts (OCs) (25). Compared with OCs, more *in vitro* studies have focused on the effects of Lf on OBs. Nagashima et al. found that human recombinant Lf promotes MC3T3-E1 cell differentiation and calcification (26). Another study indicated that Lf mediates the enhanced osteogenesis of adipose-derived stem cells (27). Mechanistically, the mitogen-activated protein kinase (MAPK) signaling pathway (28) and BCL2-Beclin1 signaling-mediated autophagy (29) participate in OB formation. In contrast, Owen et al. compared the anabolic effects of five compounds on OBs. No effect on osteogenic differentiation was observed, and even a high dose of Lf (1 mg/ml) produced an adverse effect (30). Furthermore, certain Lf-derived peptide fractions (fragment residues 624 to 632, also called LPF-C, and amino acids 97–122 from the N-terminus) also induce OB proliferation (31, 32), which warrants further investigation.

Some studies have indicated that Lf not only promotes OBs but also inhibits OC development (33–35). However, Lf does not alter bone resorption in calvarial organ culture, which suggests that Lf does not affect mature OC function (34). In other words, Lf is able to affect immature OCs but not mature ones. Lorget et al. found that Lf inhibited osteoclastogenesis and bone resorption through a mechanism independent of the osteoprotegerin/receptor activator of nuclear factor κB ligand/receptor activator of nuclear factor κB (OPG/RANKL/RANK) (36).

Many factors might lead to discrepancies in the effects of Lf on OBs or OCs, including dose, source, and intervention time. Additionally, cell type might also be an important reason. For example, Lf at the same dose could promote differentiation and calcification in MC3T3-E1 cells (26) but not in human mesenchymal progenitor-derived OBs (30). Iron saturation might further play a role, since some researchers observed that the biological effects of Lf varied with iron saturation levels in other diseases (37). Meanwhile, we should hold a cautious attitude toward the results from the cell-based studies due to the inherent limitations in their evidence hierarchy.

## Effects of the Lf forms on bone

Most studies on Lf have primarily focused on the bovine or human form. Generally, they appear to exhibit comparable activity (38); however, it should be emphasized that the activities are not always interchangeable, because their modes of intestinal receptor recognition is inconsistent (39).

Structure-function relationship studies suggest that the differences are minimal for the effects of the various Lf forms on osteogenic activity (40). The iron saturation level of Lf is not a key factor affecting OB function or mitogenic activity in MC3T3-E1 cells (41). In addition, the glycosylated forms and source of Lf do not alter its mitogenic activity (42). Although Wang et al. found that the osteogenic activity of Lf decreased with increasing iron saturation (43), and Zhang et al. found that bovine Lf appears to have more proliferative capacity compared with human Lf (44), the differences may be minor. Studies on Lf in OCs are relatively insufficient, whereas its osteoclastogenic activity appears to be located in the N-lobe of recombinant Lf (38).

## Potential mechanisms underlying the effects of Lf on OP

Numerous studies have examined the underlying mechanisms of Lf in OP, as summarized in Table 1. However, many conclusions remain speculative, and the exact mechanisms are poorly understood. A lack of high-quality studies also is an issue. A comprehensive exploration on the mechanisms is necessary for the applications of Lf. Emerging technologies such as spatial metabolomics and single-cell sequencing offer new opportunities for mechanism exploration. Meanwhile, although many pathways are involved in the protective effects of Lf in OP, regulation of the balance between osteoblasts and osteoclasts remains a fundamental mechanism.

**Table 1:** Potential mechanisms of Lf on OP.

**Type**	**Subject**	**Model**	**Intervention**	**Effects and/or mechanisms**
*In vitro* (34, 42)	Bone-forming cells Osteoblasts Cartilage cells		0.1 μg/ml	Proliferation increase
	Murine bone marrow culture system Calvarial organ culture		> 1 μg/ml	Decrease osteoclast development, but does not influence mature osteoclast function
*In vivo* (34, 42)	Adult male mice		local injection (0.04, 0.4, and 4 mg)	Increase calvarial bone growth
*In vivo* (124)	Female SD rats	OVX	oral administration (0.85, 8.5, 85 mg/kgbw)	Protect against BMD loss Improve bone microarchitecture Reduce TNF-a and IL-6 Elevate calcitonin
*In vivo* (46)	Female SD rats	OVX	10–2,000 mg/(kg·d)	Preserve bone mass and improve bone microarchitecture Enhance bone formation, reduce bone resorption, and decrease bone mass loss Suppress RANKL/OPG mRNA ratio
*In vivo* (45)	Female BALB/c mice	OVX	2, 20, and 100 mg/(kg·d)	Improve BMD Suppress RANKL/OPG ratio Regulate osteoimmunology pathway
*In vitro* (28)	ME3T3-E1		20–500 μg/ml	Promote dose-dependently cell proliferation Stimulate MAPK signaling pathways
*In vivo* (48)	Male SAMP6 mice		Oral administration 2 g/(kg·d)	Improve bone mass and microstructure Increase Igf1 mRNA expression and activate AKT Decrease Rankl/Opg mRNA Decrease the expression levels of p16 and p21
*In vivo* (47)	Female SD rats	OVX and fracture	Oral 85 mg/(kg·d)	Not only accelerate bone growth at an early stage of OPF healing, but also shortens the remolding process Increase BALP in serum Decrease TRAP5b and TNF-a in serum Lower RANKL/OPG mRNA ratio in callus
*In vitro* (30)	hES-MP cells		0.01–1 mg/ml	Reduce metabolic activity and cell number Decrease ALP activity and mineral deposition
*In vitro* (31)	MC3T3-E1		Lf-derived peptide	promote ALP activity and calcium deposition bind to EGFR to activate the MAPK pathway
*In vitro* (32)	MC3T3-E1		Lf-derived peptide 1–1,000 μg/ml	Promote osteoblast proliferation and ALP activity
*In vitro* (29)	Primary osteoblasts		1, 10, and 100 μg/ml	Inhibit BCL2 expression and further enhance Beclin1-dependent autophagy activation
*In vitro* (54)	Primary rat osteoblasts		10 and 100 μg/ml	Inhibit apoptosis
*In vitro* (53)	Primary rat osteoblasts		1–1,000 μg/ml	Promote osteoblast proliferation and inhibit apoptosis through IGF-1R
*In vitro* (53)	MC3T3-E1		100 μg/ml	Stimulate osteoblast differentiation mainly through LRP-1-independent PKA and p38 signaling pathways

### OPG/RANKL/RANK signaling pathway

The imbalance between OBs and OCs is a key factor in OP pathogenesis. The OPG/RANKL/RANK system plays an important role in this process. RANKL is expressed by OBs, and it can activate its receptor (RANK) expressed on OCs to promote OC formation. Moreover, the secretory glycoprotein OPG inhibits the effects of RANKL as a decoy receptor. Thus, abnormal alterations in the RANKL/OPG ratio may increase bone resorption and decrease bone formation.

There was direct evidence from animal studies to support that Lf could protect against OP via this signaling pathway. In an estrogen-dependent bone loss model, Fan et al. reported that Lf administration increases BMD in ovariectomized (OVX) female mice, accompanied by a decrease in the RANKL/OPG ratio (45). Similar results were observed in OVX rat models (46, 47). Moreover, the lower RANKL/OPG ratio may be a result of upregulation of IFN-γ, IL-5, and IL-10 (45). Chen et al. also reported that the ameliorative effects of Lf on aging-suppressed osteogenesis through IGF-I signaling were associated with an increased OPG/RANKL ratio at the mRNA level in SAMP6 mice (48).

### IGF1 signaling pathway

Insulin-like growth factor 1 (IGF1) is a major mediator of skeletal growth (49). Aging is a major risk factor for OP, and IGF1 may play a crucial role in the development of aging-related OP (50). There is also direct evidence to support the role of IGF1 in the effects of Lf on OP. Chen et al. examined the effects of Lf in a senile OP model (SAMP6 mice) and in senescent OBs (48). The results indicated that Lf could improve bone metabolism and increase *Igf1* mRNA expression *in vivo*. Moreover, Lf improved OB proliferation in an *in vitro* senescence model (48). Several studies have demonstrated that aging results in oxidative stress in the body, which may contribute to senile OP (51, 52). Lf treatment could inhibit oxidative stress and delay senescence by decreasing p16 and p21 expression levels. Further, knockdown of *Igf1* attenuated the effect of Lf on osteogenesis (48), which enhanced the causal inference reliability. Lf-mediated IGF1 upregulation may play a more important role in age-related OP compared with other molecules. Lf may also inhibit apoptosis to promote osteogenesis by upregulating IGF1/IGF1R *in vitro* (53, 54). Furthermore, knockdown of the IGF1 gene or silencing of IGF1R increases apoptosis in OBs (53, 54). Interestingly, Lf exhibited higher PI3K and RAS phosphorylation levels in IGF1R-silenced OBs, suggesting that Lf might activate PI3K and RAS through an IGF1R-independent pathway (53). Overall, current evidence suggested that Lf may upregulate IGF1 to influence its downstream pathway and directly activate IGF1.

### Autophagy

Autophagy is an evolutionarily conserved intracellular “self-eating” process that contributes to the onset and progression of osteopenia and OP. Aging, estrogen deficiency, and high-fat diets can trigger the adipogenic differentiation of mesenchymal stem cells (MSCs) and BMD reduction (55). In addition, the activation of autophagy is correlated with the osteogenic differentiation of MSCs (56). Estrogen can inhibit apoptosis induced by serum deprivation of osteoblasts, which may be partly achieved by promoting autophagy (57). Autophagy also plays a role in signaling pathways, which is significant to osteogenesis. For example, autophagy upregulation is considered one reason for the IGF1-simulated osteogenic differentiation of osteoblasts (58). Direct evidence indicates that Lf can inhibit B-cell lymphoma 2 (BCL2) expression in osteoblasts, further enhancing Beclin1-dependent autophagy activation, which may positively influence osteoblast formation (29). To further investigate the role of BLC2 in Lf-promoted autophagy in OBs, the researchers upregulated BCL2 expression, and it reversed the Lf-induced autophagy promotion. A similar phenomenon also occurred after Beclin1 silencing (29). Regrettably, all data were obtained from *in vitro* studies. This gap necessitates future validation through well-designed animal experiments.

### Bone morphogenetic protein signaling pathway

Bone morphogenetic proteins (BMPs) are cytokines belonging to the transforming growth factor-β (TGF-β) superfamily (59). In particular, BMP2 is considered the gold standard for bone regeneration (60), and it is an osteogenic factor approved by the FDA for clinical use (61). Mouse models generated by suppressing BMP signaling in OBs exhibit osteopenia phenotypes (62–64), further demonstrating the osteogenic role of BMPs in promoting OB differentiation. However, hyperactivated BMP signaling is a risk factor for heterotopic ossification, which is a major side effect of BMP treatment (65). At the molecular level, although the mechanisms were not fully elucidated, there are several potential pathways for promoting osteogenesis by BMPs (59): (1) positively regulating Runx2; (2) crosstalk between BMP and WNT signaling; (3) inducing the expression of osteogenesis-related transcription factors; and (4) positively regulating mammalian target of rapamycin (mTOR) activity. The low-density lipoprotein receptor-related protein (LRP) may also be a receptor for both WNT and Lf (66, 67). BMP signaling appears to have dual effects on bone formation, manifested as antagonizing osteogenesis in OB progenitors, negatively regulating mineralization, and collagen maturation (68–70). This may be the result of WNT antagonist expression induced by the BMP receptor and reduced β-catenin activation (71, 72). Li et al. hypothesized that BMP antagonizes bone formation by inhibiting WNT/β-catenin signaling (59). Alternatively, BMP signaling may promote OC differentiation (59). Nonetheless, the sophisticated interactions between BMPs and WNT/β-catenin and the relationships of BMPs to OB-OC coupling warrant further exploration.

The direct effects of Lf on BMPs were not reported; however, indirect evidence suggests regulatory effects of Lf on the BMP signaling pathway. For example, Lf hydrolysate from the N-lobe promoted OB differentiation in a BMP-dependent manner *in vitro*, and it could promote osteogenic effects through increasing BMP2 production in OVX rats (73). However, it has not been confirmed that the Lf segment can be generated in the digestive tracts and absorbed into the blood. It remains unknown whether orally administered Lf can exert its bioactivity in this manner.

### Liver–bone axis

The liver is the central metabolic organ of the body, and it plays an important role in bone homeostasis. Approximately 40% of individuals with OP have other chronic conditions, including chronic liver injury. The physical distance between the liver and bone limits their direct interaction; however, the liver can communicate the bone by secreting signaling molecules. Lu et al. reported that dysregulation of the liver–bone axis promoted the progression of hepatic osteodystrophy (74). In the liver–bone axis, hepatokine lecithin-cholesterol acyltransferase (LCAT) promotes reverse cholesterol transport from the bone to the liver, whereas its loss may exacerbate the bone loss phenotype. Many studies have described the protective effects of Lf against liver injury (75), and our studies have also shown that Lf can prevent ethanol-induced liver injury in mice (76–78). Although no direct evidence has been established, the present data suggest that the liver–bone axis may be a mechanism for the protection of Lf against OP.

### Gut microbiota

Although there was no direct evidence to support that the effects of Lf on OP depend on the gut microbiota, interactions between the gut microbiota and OP have recently been a subject of interest for researchers (79, 80). Gut microbiota dysbiosis has been observed in patients with OP (81–83). However, studies using germ-free or antibiotic-treated mice have produced conflicting results regarding the effects of gut microbiota on bone (84–87). The results were questioned by some scholars, who argued that data from germ-free animals may not be applicable to individuals with normal gut microbiota, and that the unintended effects of antibiotics could not be avoided (80). Understanding the complex association between gut microbiota and OP remains challenging. Moreover, fecal microbiota transplantation is not considered an effective option for the treatment of OP because of the harmful bacteria present in the transplant material (80). Therefore, supplementation with one or several probiotics may be a feasible strategy. *Lactobacillus* and *Bifidobacteria* are two conventional probiotics, and several studies have confirmed their beneficial effects on bone remodeling (88–93). In addition, *Akkermansia*, as a representative of “next-generation probiotics,” also exhibited a positive effect on BMD (94, 95). However, these findings are mainly derived from preclinical studies. Nilsson et al. conducted a well-designed trial to assess the effects of *Lactobacillus* on bone loss (88). However, this trial included only older women, thus limiting the generalizability to the general population. Moreover, the small sample size also limited the reliability of the findings.

Theoretically, a “Lf-gut microbiota-metabolites-bone” regulatory axis may exist. The aforementioned three bacteria are closely associated with Lf supplementation. An increased abundance of *Lactobacillus* has been observed in individuals treated with Lf (96). The growth-promoting effects of Lf on *Bifidobacteria* have also been reported (97). Interestingly, the pepsin hydrolysate of bovine Lf showed stronger bifidogenic effects than natural bovine Lf on some strains of *Bifidobacteria* (98). Thus, Lf peptides may represent the active bifidogenic form of Lf (97). Furthermore, bifidogenic effects may be achieved by Lf-binding proteins localized at the poles of bifidobacterial cells (99). Furthermore, oral Lf may increase *Akkermansia* abundance in the gut microbiota (100). This was also confirmed in our experiments (76, 78). The regulation of gut microbiota on bone was likely mediated through their metabolites, of which short-chain fatty acids (SCFAs) were widely recognized as an important candidate (101). Among SCFAs, propionate can only be generated by a few specific bacterial strains (102). Coincidentally, *Akkermansia* can generate propionate (103), which might be a mechanistic reason for the effects of Lf on OP. While germ-free or antibiotic-treated mice may not represent optimal models, they remain an appropriate choice to verify the relationship among Lf, OP, and gut microbiota. Although the crosstalk between gut microbiota and bone has been reported (80, 101), the influence of Lf on OP through gut microbiota remains theoretical and requires further evidence.

## Lf peptides

Lf is nearly completely degraded in the stomach; however, some fragments resist further digestion (104, 105). Therefore, the biological activity of Lf may depend on its peptide fragments. Lactoferricin (Lfcin) and lactoferrampin (Lfampin) are two Lf fragments of interest that exhibit antimicrobial effects (106). Moreover, the anticancer and immunomodulatory effects of Lfcin have been reported (107, 108). Whether Lfcin or Lfampin influences bone metabolism has not yet been established.

LFP-C (FKSETKNLL) is a peptide from bovine Lf hydrolysates generated through pepsin digestion. Its osteogenesis activity was demonstrated *in vitro* (31). Molecular docking suggested that the osteogenesis of LFP-C may result from its binding to the key domain (Lys13-Thr15-Gln16-Leu17-Gly18-Asp22) of the epidermal growth factor receptor (EGFR), which activates the MAPK pathway; however, biological validation has not been performed (31).

The LP2 peptide (RKVRGPPVSCIKRDSPIQ) from human Lf has self-assembly properties and skeletal bioavailability. LP2 stimulates OB differentiation through a BMP-dependent mechanism and osteoblastic production of OPG. Moreover, the subcutaneous administration of LP2 accelerates bone healing and bone formation i*n vivo* (73); however, the underlying molecular mechanisms were not examined. Because of the low resistance of Lf to digestion, Lf peptides may exhibit a higher simulation effect than Lf, particularly *in vitro*. Additionally, the active form of Lf may be its digestive hydrolysate rather than the intact molecule. Therefore, using intact Lf for *in vitro* experiments may not fully simulate the real *in vivo* effects, and the peptides may have higher research value for exploring the underlying mechanisms of Lf.

## Optimal Lf doses for humans

The main source of Lf is milk in the daily diet. In milk, the Lf contents mainly concentrate on 0.1~0.2 mg/ml (109, 110). Thus, an adult can ingest 50-100 mg per day through a regular diet. In a rat study, oral administration of Lf at 2 g/kgBW/d (equivalent to 20 g/d for an adult) for 13 weeks did not produce adverse effects (111). Another study also found that a daily intake of up to 9 g Lf is safe for humans (112). Due to its proven safety, Lf has been approved to be added to infant formula in many countries (113), and FAO and WHO recommend the level of Lf supplementation is 500 mg/kg in infant formula (114). Moreover, for adults, an expert consensus indicated that the recommended daily supplementation of Lf is 200–600 mg (115). It should be noted that these doses are intended for the general population. To date, there is still no evidence-based recommended Lf dose for OP prevention and treatment. Although some studies indicated that excessive doses of Lf may have potential negative effects (78, 116), this concern is likely of limited practical relevance. Due to the high cost of Lf supplements, excessive intake is virtually impossible in the real world.

## Clinical applicability of Lf

So far, most evidence for the beneficial effects of Lf on bone metabolism derives from preclinical studies, and the clinical trials are markedly lacking. Although no clinical trial has directly focused on Lf and OP, one randomized controlled trial investigated milk ribonuclease-enriched Lf on bone turnover markers. In this trial, milk ribonuclease-enriched Lf supplementation displayed positive effects on serum bone turnover biomarkers in postmenopausal women aged 40–60 years (16). However, it cannot be confirmed that the changes were attributed to Lf; meanwhile, the study was performed in a special population, and BMD was not determined, which might limit its generalizability and reliability. Despite these limitations, Lf is still remains a promising agent for OP protection due to its broad biological activities and high safety (5, 117). In the future, large-scale randomized controlled trials are required to validate the efficacy of Lf in OP. Of course, Lf, as a natural food component, is not as therapeutically effective as conventional pharmaceuticals. Lf may serve as a preventive agent or adjunctive therapy rather than a primary therapeutic agent in clinical practices.

## Lf-based nanoformulations or biomaterials

Due to the poor oral bioavailability of Lf, some groups have developed different formulations for bone health. Lf-embedded type 1 collagen membranes retain their pro-calcification effects during osteogenic differentiation *in vitro* (118). Injectable scaffolds are also considered efficient Lf delivery systems. Kim et al. reported that Lf-loaded porous polymicrospheres promote osteogenic differentiation by controlling Lf release (119). Liposomes are another important delivery system (120, 121). Recently, these topics have been thoroughly reviewed by other scholars (122, 123) and are beyond the scope of this review. Therefore, we have not discussed them in detail in this review.

## Conclusion and future outlook

**Figure 1:**
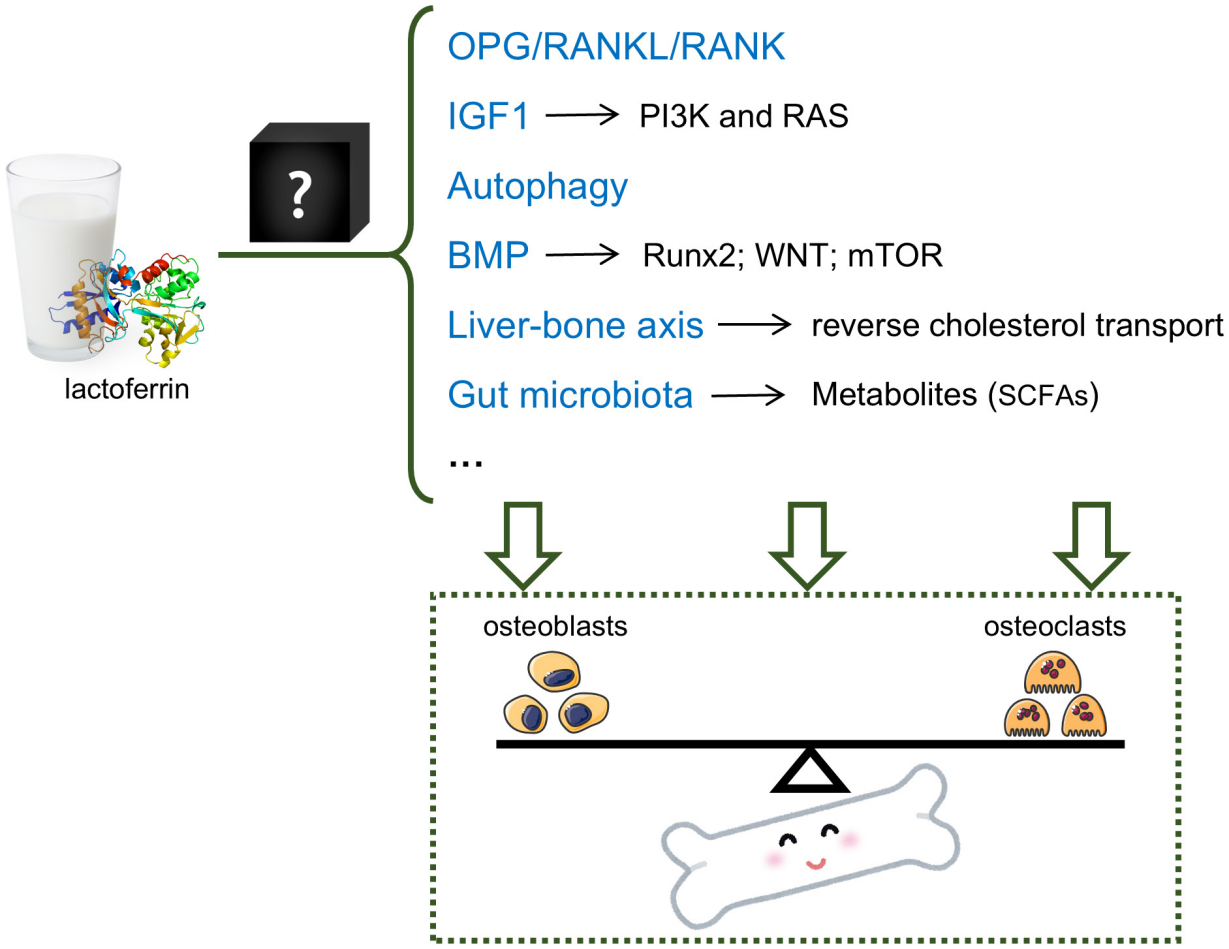
Potential mechanisms of the OP-protective effect of lactoferrin. Lactoferrin may regulate the balance between osteoblasts and osteoclasts via multiple mechanisms, including but not limiting OPG/RANKL/RANK pathway, IGF1 pathway, autophagy, BMP pathway, liver-bone axis and gut microbiota.

Several mechanisms have been reported to explain the effects of Lf on OP (Figure 1); however, these mechanisms are primarily derived from cell or animal experiments. The lack of clinical evidence remains the largest pain point for the applications of Lf. It is valuable to conduct a randomized, placebo-controlled, double-blinded trial to assess the efficacy of Lf. In this trial, BMD is a more valuable outcome besides serum biomarkers such as ALP, β-CTx, and TP1NP. Considering that the immediate effects of Lf (as a natural food component) may be weak, a long-term intervention is recommended. And other proteins without medical effects can be selected as a placebo. It should be noted that we need to maintain a “cautiously” optimistic attitude until the effectiveness of Lf on OP is confirmed. Moreover, the mechanisms through which lactoferrin regulates these pathways remain a “black box,” and deconstructing this box is a research topic that warrants further exploration.
